# Anti-GBM-Nephritis

**DOI:** 10.1007/s11560-023-00666-2

**Published:** 2023-06-13

**Authors:** Martin Windpessl, Andreas Kronbichler

**Affiliations:** 1 Abteilung für Innere Medizin IV, Bereich Nephrologie, Klinikum Wels-Grieskirchen, Grieskirchner Str. 42, 4600 Wels, Österreich; 2 Abteilung für Innere Medizin IV, Nephrologie und Hypertensiologie, Medizinische Universität Innsbruck, Anichstr. 35, 6020 Innsbruck, Österreich

**Keywords:** Anti-GBM-Erkrankung, Goodpasture, Nierenversagen, Cyclophosphamid, Imlifidase, Anti-glomerular basement membrane disease, Goodpasture, Renal insufficiency, Cyclophosphamide, Imlifidase

## Abstract

Antikörper gegen die glomeruläre Basalmembran (GBM) verursachen eine aggressiv verlaufende Autoimmunerkrankung, die den Kleingefäßvaskulitiden zugeordnet wird und im weiteren Sinn auch als Goodpasture-Syndrom bekannt ist. Sie verläuft entweder auf die Nieren beschränkt (Anti-GBM-Nephritis) oder manifestiert in Form eines pulmorenalen Syndroms (Anti-GBM-Erkrankung). Im Laufe der letzten Jahre hat sich das Krankheitsspektrum erweitert. Insbesondere die „doppelt positive“ serologische Konstellation, also die Koexistenz von Anti-GBM- und antineutrophilen zytoplasmatischen Antikörpern (ANCA), wurde klarer abgegrenzt, was auch therapeutische Implikationen nach sich zieht. Ein rascher Behandlungsbeginn ist essenziell, um die Prognose entscheidend zu verbessern. Die Plasmapherese bleibt eine zentrale Therapiesäule mit dem Ziel, pathogene Autoantikörper zu entfernen. Es sind nun bessere klinische und histologische Merkmale definiert, die es erlauben, frühzeitig den Nutzen einer etwaigen Therapiefortsetzung bezüglich Nierenfunktion einzuschätzen. Dieser Artikel gibt einen Überblick über neue Erkenntnisse hinsichtlich Krankheitsverlauf („atypische“ Varianten) und setzt den Schwerpunkt auf klinisch relevante Aspekte in der Diagnostik und insbesondere auf neue Therapieansätze. Mit Imlifidase zeichnet sich eine vielversprechende Erweiterung der Behandlungsmöglichkeiten ab.

## Abstract (en)

Autoantibodies against the glomerular basement membrane (GBM) cause an aggressive form of small vessel vasculitis, previously also referred to as Goodpasture syndrome. The disease either runs a limited course in the kidneys (anti-GBM nephritis) or manifests as pulmonary renal syndrome (anti-GBM disease). Over the past decade, a broader range of manifestations has been recognized. In particular, double positive serology, i.e., the coexistence of anti-GBM autoantibodies and antineutrophil cytoplasmic antibodies (ANCA), has been delineated in more detail, which also has treatment implications. Urgent treatment is essential to decisively improve outcomes. Plasma exchange remains a central component of current treatment strategies, with the overall aim to remove pathogenic autoantibodies. Better clinical and histological markers have now emerged, enabling an early stratification of patients who will benefit from continuous immunosuppressive therapy in terms of the renal prognosis. This article provides an overview of novel insights into the disease course (including atypical variants), with a focus on novel clinically relevant aspects in the diagnostics and particularly in new treatment approaches. Imlifidase has emerged as a promising extension to the treatment options.

## Einleitung

Die Anti-GBM(glomeruläre Basalmembran)-Nephritis ist eine oft aggressiv verlaufende, potenziell lebensbedrohliche Erkrankung, die gemäß der Chapel-Hill-Klassifikation den Kleingefäßvaskulitiden zugeordnet wird [16]. Als namensgebende Besonderheit finden sich im Serum Antikörper gegen Bestandteile der GBM, konkret gegen die nichtkollagene Domäne (NC1) der Alpha-3-Kette von Typ-IV-Kollagen (α3[IV]NC1). Dieses Zielantigen findet sich außer in der GBM auch in der Basalmembran der Lungenalveolen, was das Manifestationsspektrum erklärt [9].

Mitte des 20. Jahrhunderts wurde von Stanton und Tange das Eponym Goodpasture-Syndrom geprägt [27]. Sie charakterisierten anhand der Krankengeschichte von 9 jungen Patienten akribisch ein dramatisches Krankheitsbild, das bei allen Betroffenen rasch zum Tod führte (4 Patienten verstarben noch am Aufnahmetag an einer Lungenblutung). Die Entität erinnerte die Autoren an eine Publikation von Ernest Goodpasture aus dem Jahr 1919, in welcher 2 Patienten beschrieben wurden, die ebenfalls an einer Lungenblutung verstorben waren (bei 1 der beiden war in der Obduktion auch eine Glomerulonephritis [GN] nachweisbar; [4]). Goodpasture schrieb dies der gegen Ende des Ersten Weltkriegs grassierenden Spanischen Grippe zu. Mittlerweile geht man davon aus, dass es sich dabei um eine andere Form einer Vaskulitis gehandelt hat (vermutlich um eine ANCA[antineutrophile zytoplasmatische Antikörper]-assoziierte Vaskulitis [AAV]; [24]).

Es wird zwischen einer pulmorenalen Manifestation und einer renal-limitierten Form unterschieden

Anhand der klinischen Präsentation wird zwischen einer pulmorenalen Manifestation (Goodpasture-Syndrom im engeren Sinn) und einer renal-limitierten Form unterschieden. Allerdings ist die Nomenklatur im Wandel, und es setzt sich für die auf die Nieren beschränkte Verlaufsform zusehends der Begriff Anti-GBM-Nephritis durch, während bei zusätzlicher Lungenbeteiligung von Anti-GBM-Erkrankung gesprochen wird [16].

Es handelt sich um eine seltene Entität. In Europa liegt die Inzidenz bei etwa 1 Fall pro Million Einwohner und Jahr [25]. Die Krankheit zeigt 2 Häufigkeitsgipfel, und zwar zwischen dem 20. und 30. Lebensjahr sowie in der 6. und 7. Lebensdekade; sie kann aber in jedem Alter auftreten, wobei Manifestationen im Kindesalter äußerst ungewöhnlich sind [16]. Eine saisonale Variabilität spricht für einen Zusammenhang mit (Atemwegs‑)Infektionen [16]. Während der COVID-19(„coronavirus disease 2019“)-Pandemie erschienen mehrere Publikationen, die über ein Neuauftreten einer Anti-GBM-Erkrankung im Rahmen einer SARS-CoV-2(„severe acute respiratory syndrome coronavirus 2“)-Infektion berichteten [21]. Außerdem ist neben einem zeitlichen auch eine örtliche Clusterbildung belegt [2].

Ein Zusammenhang zwischen pulmonaler Manifestation mit Nikotinexposition sowie mit anderen pulmonalen Noxen ist seit Jahrzehnten fest etabliert [16]. Des Weiteren werden genetische Faktoren, insbesondere eine Assoziation mit bestimmten HLA(humanes Leukozytenantigen)-Antigenen, in der Krankheitsentstehung impliziert. Gleichwohl gibt es nur wenige Berichte über familiäre Formen [6].

Die Anti-GBM-Erkrankung ist ein Paradebeispiel für eine Autoimmunkrankheit. Zirkulierende Antikörper mit definiertem Zielantigen (Epitop α3[IV]NC1 = „Goodpasture-Antigen“) schädigen entsprechende Organstrukturen durch Komplementaktivierung und Rekrutierung von Entzündungszellen [18, 25]. Die zentrale Bedeutung der Autoantikörper in der Pathogenese gilt seit einem klassischen Tierexperiment als gesichert, bei dem es gelang, Autoantikörper von Patienten mit Goodpasture-Syndrom auf Primaten zu übertragen und dadurch eine GN auszulösen. Üblicherweise handelt es sich um IgG(Immunglobulin G)-Antikörper (meist IgG1- und IgG3-Subklasse; [16, 22]). Die Höhe der Antikörper hat auch prognostische Aussagekraft [25]. Neben α3(IV)NC1 wurden rezent auch andere Bestandteile der GBM als Zielantigene beschrieben (Peroxidasin und Laminin-521; [13]). Bevor der Stellenwert dieser Publikationen abschließend beurteilt werden kann, bedarf es einer Validierung in unabhängigen Untersuchungen. Eine detaillierte Darstellung der Krankheitsentstehung erfolgte hier in einer früheren Publikation zu dieser Thematik [17]. Bezüglich neuer pathophysiologischer Erkenntnisse sei auf rezente Übersichtsarbeiten verwiesen [13, 25].

## Nur die Hälfte der Patienten weist eine pulmonale Beteiligung auf

Jüngere Patienten manifestieren typischerweise mit dem Bild eines pulmorenalen Syndroms. Konstitutionelle Symptome können der Manifestation mitunter Wochen vorangehen, was die Diagnosefindung erschwert. Ältere Patienten zeigen hingegen häufiger eine renal-limitierte Manifestation [5]. Ein isolierter pulmologischer Verlauf stellt eine Rarität dar, trotzdem muss bei einer diffusen alveolären Hämorrhagie (DAH) auch ohne Nierenschädigung stets an eine Anti-GBM-Erkrankung gedacht werden [25]. Die respiratorischen Symptome reichen von trockenem Husten, Belastungsdyspnoe bis zum Vollbild einer akuten Lungenblutung mit massiven Hämoptysen, ihr Fehlen schließt eine klinisch relevante DAH allerdings nicht aus. Bei etwa 4 % aller Patienten, die aufgrund einer DAH eine intensivmedizinische Behandlung benötigen, liegt eine „pure“ Anti-GBM-Erkrankung vor, eine „doppelt positive“ Konstellation, also eine Koexistenz von Anti-GBM-Antikörpern mit ANCA, findet sich in diesem Kontext bei etwa 14 % ([3]; diese Konstellation wird weiter unten ausführlicher beschrieben).

Bei einer diffusen alveolären Hämorrhagie muss stets an eine Anti-GBM-Erkrankung gedacht werden

Die rapid-progressive GN (RPGN), gekennzeichnet durch eine rasche Abnahme der Nierenfunktion, ein nephritisches Harnsediment und das histologische Korrelat von Halbmonden, ist der typische renale Verlauf [24]. Von den 3 häufigsten Ursachen einer RPGN im Erwachsenenalter (AAV, Immunkomplex-GN und Anti-GBM-GN) ist letztere zwar am seltensten (etwa 15 % einer älteren Chapel-Hill-Fallserie [*n* = 632] zufolge), zeigt jedoch die schlechteste Prognose [12]. Hyperakute Spielformen können binnen weniger Wochen im Vollbild eines anurischen Nierenversagens gipfeln. Die Erkrankung verläuft meist monophasisch („one and done“; [11]). Rezidive kommen selten vor und sind dann meist Ausdruck fortwährenden Rauchens. In einer großen monozentrischen Kohorte (Hammersmith Hospital, UK; *n* = 71) wurden nach erfolgreicher Behandlung (und vorübergehender Seronegativität) 2 Rezidive berichtet [15]; diese können auch erst nach Jahren auftreten.

Die Autoantikörperproduktion sistiert nach Krankheitsausbruch spontan. In einer älteren Fallserie mit bei Aufnahme dialysepflichtigen Patienten ohne pulmonale Manifestation (*n* = 8) wurden die Antikörper im Mittel nach 11 Monaten negativ, ohne dass die Patienten eine Therapie erhielten [7].

Auch eine Fall-Kontroll-Studie von Olson et al. trug zum besseren Verständnis des natürlichen Verlaufs der Erkrankung bei [20]. Zum Zeitpunkt des Dienstantritts (und in der Folge 2‑jährlich) werden von allen Personen der US-Streitkräfte Serumproben in einer Biobank gespeichert. Die Autoren analysierten retrospektiv Seren von 30 Patienten dieser Kohorte, die später eine Anti-GBM-Erkrankung entwickelten. Während 4 dieser Patienten (13 %) positive Antikörper aufwiesen, war die Serologie in der Kontrollgruppe bei allen negativ. Die durchschnittliche Zeit zwischen Blutabnahme und Krankheitsmanifestation betrug 195 Tage (Spannweite: 4–1346). Fast alle dieser Patienten wiesen präsymptomatisch auch positive ANCA auf.

## Die Diagnose fußt auf dem Nachweis der Autoantikörper und dem histologischen Nachweis linearer Ig-Depots

Erhöhte Entzündungswerte, Nierenfunktionseinschränkung sowie eine bisweilen ausgeprägte Anämie als Ausdruck einer chronisch-rezidivierenden Lungenblutung sind typische, aber unspezifische Laborbefunde. Im Harnteststreifen findet sich regelhaft eine Hämaturie (mitunter berichten Patienten von Makrohämaturie), eine Proteinurie ist überwiegend subnephrotisch (Ausnahme: seltene Sonderform in Assoziation mit einer membranösen Nephropathie; [9]). Im Lungenröntgen können als Ausdruck der DAH diffuse, bilaterale (vereinzelt auch asymmetrische) Verschattungen vorliegen [24].

Diagnostisch wegweisend ist der Nachweis zirkulierender Anti-GBM-Antikörper [24]. Gleichzeitig muss stets eine Testung auf ANCA erfolgen – einerseits, weil sich eine AAV klinisch nicht von einer Anti-GBM-Erkrankung unterscheiden lässt, andererseits, weil bei etwa einem Drittel dieser Patienten auch ANCA nachweisbar sind [16, 26]. Meist handelt es sich dabei um ANCA vom MPO(Myeloperoxidase)-Subtyp. Solche „doppelt positiven“ Patienten unterscheiden sich im Verlauf von Patienten mit „einfacher“ Serologie. Die Akutmanifestation entspricht zumeist der klassischen Anti-GBM-Erkrankung, das zusätzliche Vorhandensein von ANCA bringt im Verlauf ein signifikantes Rezidivrisiko mit sich (analog zur AAV). Entsprechende therapeutische Implikationen werden weiter unten dargestellt.

## In seltenen Fällen gelingt kein Nachweis von Autoantikörpern im Serum

Zum Nachweis zirkulierender Autoantikörper kommen entweder Chemilumineszenz- (ChLIA) oder Enzymimmunassays („enzyme-linked immunosorbent assay“, ELISA) zum Einsatz. Eine Studie, in der Proben von 67 Patienten mit Anti-GBM-Erkrankung und 221 Kontrollpatienten analysiert wurden, verglich beide Optionen. Während alle 67 Proben durch den ChLIA-Test als positiv detektiert wurden (Sensitivität: 100 %), wurden nur 60 Proben durch den ELISA als positiv erfasst (Sensitivität: 89,6 %). Ein Nachweis von Anti-GBM-Antikörpern gelang in 3 der Kontrollproben durch den ChLIA-Test (2 Patienten mit AAV und 1 Patient mit IgA-Nephropathie; Spezifität: 98,6 %), wobei der ELISA keine positiven Resultate fand (Spezifität: 100 %; [14]). Dies bedeutet, dass beide Tests eine exzellente Diskriminierung aufweisen, wobei der ChLIA-Test sensitiver ist als der ELISA und dadurch bei mehr Patienten mit Anti-GBM-Erkrankung Antikörper nachweist.

In der Literatur finden sich einige Fallserien von entweder atypischen Fällen einer Anti-GBM-Erkrankung oder eindeutig klinischen Fällen (z. B. histologisch gesicherte Nierenbeteiligung) mit nichtdetektierbaren Antikörpern. Die meisten Tests basieren auf dem Nachweis von IgG1 der α3-Kette von Typ-IV-Kollagen. Antikörper gegen IgG4 der GBM wurden bei 4 Patienten mit entweder negativem oder grenzwertig positivem Test nachgewiesen; alle waren weiblich und hatten einen lebensbedrohlichen Verlauf mit schwerer alveolärer Hämorrhagie [19]. Weitere atypische Formen der Anti-GBM-Erkrankung präsentieren sich etwa mit Antikörpern, die gegen IgA gerichtet sind [9].

## Histologische Merkmale

**Figure 1:**
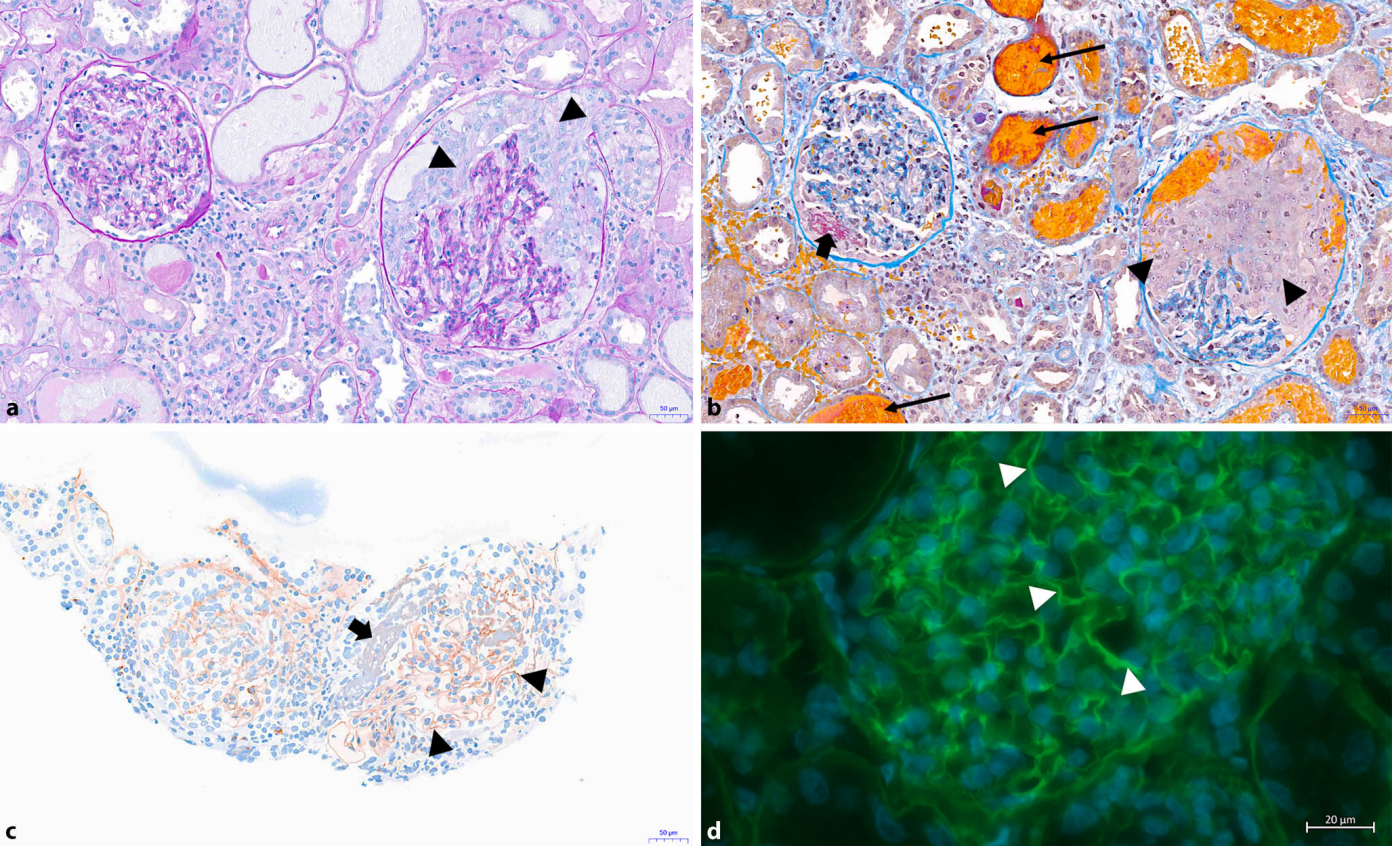

Neben der Diagnosesicherung dient die Nierenbiopsie auch der Prognoseeinschätzung und der Therapieplanung, sie sollte daher stets angestrebt werden [9]. Bei kritisch kranken Patienten ist dies allerdings nicht immer möglich. Typischerweise zeigt die Histologie eine ausgeprägte Bildung von Halbmonden (oft > 80–100 % aller Glomeruli), wobei diese im Unterschied zur AAV häufig ähnlichen Alters sind. Obwohl eine lineare Ablagerung von Ig entlang der GBM in der Immunfluoreszenz auch bei Diabetes mellitus, systemischem Lupus erythematodes (SLE) und anderen Entitäten vorkommen kann (als unspezifischer Befund), ist dies im Kontext einer ausgeprägten glomerulären Inflammation pathognomonisch für die Anti-GBM-Nephritis (Abb. 1). „Atypische“ Formen mit milderem Verlauf wurden zuletzt besser charakterisiert [18]. Histologisch finden sich dabei lineare Ig-Ablagerungen ohne diffuse Halbmondbildung (fokal bei 8 von 20 Patienten) und verschiedene histomorphologische Charakteristika (z. B. membranoproliferative GN, thrombotische Mikroangiopathie). Diese Patienten haben häufig keine zirkulierenden Antikörper.

## Ein rascher Therapiebeginn ist wichtig, um die Prognose zu verbessern

Unbehandelt führt die Anti-GBM-Erkrankung bei typischem Verlauf rasch zum Tode, eine unverzügliche und nachhaltige Eliminierung der pathogenen Antikörper ist daher essenziell. Die Therapie umfasst die Plasmapherese sowie eine Immunsuppression ähnlich der Induktionstherapie der AAV (Glukokortikoide in Kombination mit Cyclophosphamid [CYC]) zur Unterdrückung der Nachproduktion der Autoantikörper sowie zur Entzündungshemmung (Tab. 1; [16, 26]). Diese Maßnahmen verbessern das Einjahresüberleben auf über 90 % [10].

**Table 1:** 

	Standardtherapie	Alternative Therapieoptionen
Steroide	Prednisolon (1 mg/kg KG, Maximum: 60 mg), fortlaufende Dosisreduktion mit Beendigung der Therapie nach 4 bis 5 Monaten	Intravenöse Stoßtherapie mit Methylprednisolon (wird z. B. auch in der GOOD-IdeS02-Studie vorgeschrieben)
CYC/alternative Optionen	Orales CYC (2–3 mg/kg KG) Anpassung bei schlechter Nierenfunktion (25 % Reduktion bei eGFR von 45–60 ml/min/1,73 m^2^; 40 % bei eGFR von 30–45 ml/min/1,73 m^2^; 50 % bei eGFR von 15–30 ml/min/1,73 m^2^; 60 % bei eGFR < 15 ml/min/1,73 m^2^ oder Dialysepflicht) Anpassung bei Alter > 55 Jahre (Maximum: 2 mg/kg KG) Anpassung bei Leukopenie (< 4 G/l; 75 % der letzten Dosis)	Intravenöses CYC (analog dem CYCLOPS-Schema der ANCA-assoziierten Vaskulitis, 6 bis 10 Infusionen) Rituximab: 4‑mal 375 mg/m^2^ (Beachte: Gabe während Episoden der extrakorporalen Therapie reduziert die Rituximabspiegel signifikant!) Sequenzielle Therapie mit CYC und RTX
Therapeutischer Plasmaaustausch	1- bis 1,5Faches des Plasmavolumens (Maximum: 4 l) oder 60 ml/kg KG Täglich, bis Anti-GBM-Antikörper entweder nicht mehr nachweisbar oder in einem nichttoxischen Bereich Austausch gegen Humanalbumin oder gefrorenes Frischplasma (bei Blutungskomplikationen oder rezenten Interventionen)	Immunadsorption Vorteile: Das 2,5- bis 3Fache des Plasmavolumens kann problemlos ausgetauscht werden, d. h. v. a. bei Patienten mit einem Körpergewicht von ≥ 80 kg von Vorteil; effizientere Antikörperreduktion
Erhaltungstherapie	Nicht empfohlen	Bei doppelt positiven Patienten (gleichzeitiges Vorhandensein von ANCA) empfohlen, v. a. wenn PR3-ANCA detektiert wird
Imlifidase	0,25 mg/kg (Einzeldosis); nicht zugelassen (GOOD-IdeS02-Studie); Antikörper wiederum detektierbar nach 5 bis 10 Tagen (ein Plasmaaustausch könnte danach wiederum notwendig werden)	–
Prophylaxe	*Pneumocystis-jirovecii*-Prophylaxe (Trimethoprim-Sulfamethoxazol, oder Alternativen) Vitamin D/Kalzium (während Prednisolontherapie) Magenschutz (PPI oder H_2_-Blocker) Amoxicillin/Clavulansäure oder Alternativen, wenn Imlifidase Verwendung findet	–

Diese Empfehlungen beruhen auf Erfahrungsberichten, wobei es zurzeit noch keine veröffentlichten Zulassungsstudien gibt

*ANCA* antineutrophile zytoplasmatische Antikörper; *CYC* Cyclophosphamid; *CYCLOPS-Schema* 6 CYC-Infusionen, 2. und 3. Gabe im Abstand von je 2 Wochen, die weiteren 3 Infusionen im Abstand von je 3 Wochen; *eGFR* geschätzte glomeruläre Filtrationsrate; *GBM* glomeruläre Basalmembran; *KG* Körpergewicht; *PPI* Protonenpumpeninhibitoren; *PR3* Proteinase 3; *RTX* Rituximab

Zur Beurteilung des Therapieerfolgs ist eine engmaschige Verlaufskontrolle der Autoantikörper notwendig

Der Stellenwert der Plasmaseparation bei Vorliegen einer Lungenblutung oder einer nicht dialysepflichtigen Nierenbeteiligung ist seit Jahrzehnten fest etabliert [26]. Aufgrund der hohen Antikörperlast sollen die Behandlungen primär täglich erfolgen, üblicherweise für eine Dauer von 10 bis 20 Tagen. In einer großen französischen Kohorte betrug die durchschnittliche Behandlungsanzahl 13 (Spannweite: 9–17; [10]).

Zur Beurteilung des Therapieerfolgs ist eine engmaschige (initial wöchentliche) Verlaufskontrolle der Autoantikörper notwendig. Unter Behandlung werden die meisten Patienten binnen 4 Wochen seronegativ. Persistierende Antikörper nach 2 Monaten sind ungewöhnlich; in solchen Fällen sollte eine Therapieumstellung erwogen werden. Kommt es nach ursprünglichem Behandlungsansprechen zu einem „rebound“ der Autoantikörper, kann ein Wiederbeginn der Plasmapherese notwendig werden. Meist reichen dann wenige Behandlungen aus, um eine anhaltende Seronegativität zu erzielen [16].

CYC wird gemäß der Datenlage üblicherweise oral verabreicht (2 mg/kg Körpergewicht [KG]), wobei eine Dosisadaption in Abhängigkeit von Alter und Nierenfunktion sowie unter engmaschiger Blutbildkontrolle erfolgen muss. Die Behandlungsdauer ist nicht gut etabliert, beträgt aber üblicherweise 3 Monate. Wird CYC intravenös gegeben, werden Therapiestrategien aus der AAV extrapoliert (CYCLOPS-Schema: die ersten 3 Infusionen im 2‑wöchentlichen Abstand, gefolgt von zumindest 3 weiteren Infusionen alle 21 Tage; Dosis: 15 mg/kg KG mit Adjustierungen bei Nierenfunktionseinschränkung, höherem Alter und Leukopenie; siehe Tab. 1).

Glukokortikoide stellen die dritte Behandlungssäule dar. Sie werden in einer Dosierung von 1 mg/kg KG verabreicht (maximal 60 mg pro Tag) und getapert; nach 6 Wochen sollte die Dosis bei 20 mg pro Tag liegen. Ein Zusatznutzen von initialen Methylprednisolonpulsen (für 3 Tage, maximal je 1 g) ist nicht belegt und wird von Zentren mit großer Erfahrung auch nicht propagiert (Tab. 1; [9, 16]). Gerade zu Therapiebeginn, insbesondere bei schwerwiegenden Verläufen, wird dieser Therapieweg allerdings oft eingeschlagen, zumal bei (noch) inkompletter Befundlage. Auch rezente Studienprotokolle beinhalteten Steroidpulse [28].

Im Gegensatz zur AAV ist der Stellenwert B‑Zell-gerichteter Therapien (z. B. Rituximab) nicht gut etabliert. Bis 2018 lagen lediglich 22 entsprechende Fallberichte vor, seither hat sich keine substanzielle Verbesserung der Datenlage ergeben [11]. Laut KDIGO (Kidney Disease: Improving Global Outcomes) soll Rituximab therapierefraktären Verläufen vorbehalten bleiben [23]. Auch bei Rezidiven findet es Anwendung.

Falls schon zum Zeitpunkt der Aufnahme Oligoanurie oder Dialysepflicht vorliegt und sich in der Nierenbiopsie annähernd 100 % Halbmonde finden, ist die renale Prognose so schlecht, dass von einer Behandlung kein Nutzen hinsichtlich der Nierenfunktion zu erwarten ist. Eine Therapie ist dann nur bei Lungenbeteiligung indiziert. Eine retrospektive Studie (*n* = 43) zeigte den prognostischen Stellenwert der Harnmenge am Tag der Diagnosestellung sowohl hinsichtlich des renalen Outcome als auch bezüglich des Einjahresüberlebens [1]. 27 Patienten mit Oligoanurie (< 500 ml/Tag) wiesen ein Einjahresüberleben von 81 % auf, wohingegen Patienten ohne höhergradig eingeschränkte Harnmenge keine erhöhte Mortalität zeigten. Im klinischen Alltag wird bei Diagnosestellung häufig mit einer Plasmapherese begonnen und die Situation nach Vorliegen des histologischen Befunds reevaluiert [26].

Bei doppelt positiven Patienten (v. a. mit PR3[Proteinase 3]-Subtyp) ist auch eine Erhaltungstherapie angezeigt, wofür entweder Rituximab oder Azathioprin zur Verfügung steht [26].

Es sollte nachdrücklich zur absoluten Nikotinkarenz aufgerufen werden

Eine begleitende Pneumocystisprophylaxe mit Trimethoprim-Sulfamethoxazol wird während der immunsuppressiven Behandlung empfohlen [9]. Betroffenen sollte der Stellenwert von pulmonalen Noxen auch bezüglich des Rezidivrisikos erklärt und an sie nachdrücklich zur absoluten Nikotinkarenz appelliert werden.

Noch in den 2021 aktualisierten KDIGO-Leitlinien wurde festgestellt, dass sich seit Veröffentlichung der letzten Guidelines 9 Jahre zuvor keine nennenswerten Neuerungen in den Behandlungsoptionen der Anti-GBM-Erkrankung ergeben hätten [23]. Mit Imlifidase (IdeS) liegt nunmehr ein neuer, revolutionärer Behandlungsansatz vor. Es handelt sich um eine aus *Streptococcus pyogenes* gewonnene Protease, die zirkulierende und gebundene Ig rasch und spezifisch spaltet [26]. Der Wirkstoff erhielt 2018 von der European Medicines Agency (EMA) Orphan-drug-Status für die Behandlung der Anti-GBM-Erkrankung. Im Gegensatz zur Plasmapherese werden durch das Enzym auch an Gewebe gebundene Autoantikörper abgebaut. Der Wirkeintritt erfolgt sehr rasch. In einer Phase-II-Studie lagen die Antikörperspiegel bei allen 15 Probanden bereits 6 h nach Behandlung unterhalb des Referenzbereichs. Trotz schlechter Prognose zu Therapiebeginn (geschätzte [„estimated“] glomeruläre Filtrationsrate [eGFR] < 15 ml/min/1,73 m^2^ oder Verschlechterung der Nierenfunktion trotz Therapie) blieben zwei Drittel der Probanden nach einem halben Jahr ohne Nierenersatztherapie, die eGFR der restlichen Studienteilnehmer lag zwischen 19 und 59 ml/min/1,73 m^2^ [28]. Derzeit rekrutiert eine Phase-III-Studie, geplant ist der Einschluss von 50 Patienten. Eine Limitation des breiten Einsatzes der Substanz könnte der Preis des Medikaments darstellen.

## Auch zur Prognoseeinschätzung ist die Nierenbiopsie von zentraler Bedeutung

Trotz verbesserter Gesamtprognose schreitet mehr als die Hälfte der Patienten in ein dialysepflichtiges Nierenversagen fort. Rezent konnten Floyd et al. [8] in einer internationalen Kohorte (*n* = 174) zeigen, dass insbesondere ein Anteil normaler Glomeruli von weniger als 10 % und eine Dialysepflicht zum Diagnosezeitpunkt mit einer schlechten renalen Prognose einhergehen. Diese Risikofaktoren wurden bereits in einer früheren Studie (*n* = 123) identifiziert, wobei hier auch ein prominentes interstitielles Infiltrat als unabhängiger Faktor, welcher mit einer schlechten renalen Prognose korreliert, beschrieben wurde [29].

Eine Nierentransplantation darf erst erfolgen, wenn keine Autoantikörper mehr nachweisbar sind, weil sonst häufig Rezidive im Transplantat auftreten. Üblicherweise wird daher verlangt, dass der Antikörperstatus für mindestens 6 Monate negativ ist. Die Langzeitprognose nach Transplantation unterscheidet sich gegenüber anderer Ätiologien nicht [16].

Im Sinne einer Alloimmunreaktion können Patienten mit Typ-IV-Kollagen-Erkrankungen (Alport-Spektrum) nach einer Nierentransplantation Anti-GBM-Antikörper gegen das normale Kollagen im Allograft entwickeln [25]. Dies geschieht in der Regel früh und geht mit einem hohen Risiko für ein Transplantatversagen einher. Konventionelle Testsysteme können beim Nachweis der Autoantikörper versagen.

## Fazit für die Praxis

- Aufgrund ihres häufig aggressiven Verlaufs ist eine frühe Diagnose der Anti-GBM(glomeruläre Basalmembran)-Erkrankung von zentraler Bedeutung.
- Während jüngere Patienten typischerweise mit dem Bild eines pulmorenalen Syndroms manifestieren, zeigen ältere Patienten oftmals renal-limitierte Formen.
- „Atypische“ Varianten können mildere Krankheitsverläufe zeigen.
- Die Nierenprognose wird durch klinische Charakteristika und den histologischen Befund bestimmt.
- Die isolierte Anti-GBM-Glomerulonephritis rezidiviert fast nie, hingegen zeigen „doppelt positive“ Konstellationen einen hybriden Phänotyp und sind im Verlauf von einer der AAV(ANCA[antineutrophile zytoplasmatische Antikörper]-assoziierte Vaskulitis)-ähnlichen Relaps-Neigung charakterisiert. Sie bedürfen einer Erhaltungstherapie.
- Plasmapherese und Immunsuppression mit Cyclophosphamid und Glukokortikoiden bleiben die Behandlungssäulen mit dem Ziel einer raschen Eliminierung pathogener Autoantikörper.
- Mit Imlifidase zeichnet sich eine vielversprechende neue Therapieoption ab, die jedoch mit bedeutend höheren Therapiekosten als die jetzige Standardtherapie einhergeht.
- Wegen der Seltenheit der Krankheit und der therapeutischen Herausforderungen sollten Patienten an erfahrenen Zentren betreut werden.
